# Biallelic *DNAH11* Variations Cause Male Infertility with Multiple Morphological Abnormalities of the Sperm Flagellum in Humans

**DOI:** 10.1002/mco2.70210

**Published:** 2025-05-10

**Authors:** Xue Yang, Dingming Li, Yihong Yang, Guicheng Zhao, Ying Shen

**Affiliations:** ^1^ Department of Obstetrics/Gynecology Key Laboratory of Obstetric Gynecologic and Pediatric Diseases and Birth Defects of Ministry of Education West China Second University Hospital Sichuan University Chengdu China; ^2^ Department of Andrology/Sichuan Human Sperm Bank Key Laboratory of Obstetric, Gynecologic and Pediatric Diseases and Birth Defects of Ministry of Education West China Second University Hospital Sichuan University Chengdu China

1

Dear Editor,

Asthenoteratozoospermia, defined as reduced sperm motility and abnormal sperm morphology, accounts for approximately 19% of all male infertility. Multiple morphological abnormalities of the sperm flagellum (MMAF) is a severe type of asthenoteratozoospermia, characterized by a variety of sperm tail defects, including short, absent, curl, angle, or irregular sperm flagellum. MMAF is considered as a genetic disorder, with approximately 40 related genes identified so far, yet these genes account for only about 60% of human MMAF cases [1].

Motile cilia and sperm flagella share highly conserved axoneme with a “9 + 2” microtubule architecture. The inner dynein arms (IDAs) and outer dynein arms (ODAs) are two molecular motors to provide the original energy for ciliary beating and sperm motility in humans. *DNAH* family is involved in the formation of axoneme in motile cilia and sperm flagella [1]. *DNAH11* encodes an ODA protein, and mutations in *DNAH11* are known to cause primary ciliary dyskinesia (PCD) [2]. However, the role of *DNAH11* in sperm motility and morphology has rarely been investigated.

In this study, we performed whole‑exome sequencing (WES) to investigate the potential genetic causes in two unrelated infertile patients with MMAF phenotype after ruling out conventional risk factors. Remarkably, two biallelic *DNAH11* variations (NM_001277115.2) were identified, including a compound heterozygous mutation of c.6233G>A (p.Gly2078Glu) and c.9335A>G (p.Gln3112Arg) in patient A and another compound heterozygous mutation of c.6143C>G (p.Thr2048Arg) and c.10379C>A (p.Thr3460Lys) in patient B (Figure 1A). In addition, these variants were completely absent or presented at an exceedingly low frequency in the ExAC Browser (0, 0.0000352, 0.0003, 0.0003, respectively), GnomAD (0, 0, 0.0002, 0.0002, respectively), and 1000 Genomes Project databases (0, 0, 0.000399361, 0.000199681, respectively), predicted to be deleterious by bioinformatic tools, including SIFT, PolyPhen‐2, and M‐CAP. Moreover, the affected sites of theses variants are quite conserved across different species (Figure 1A).

**FIGURE 1:**
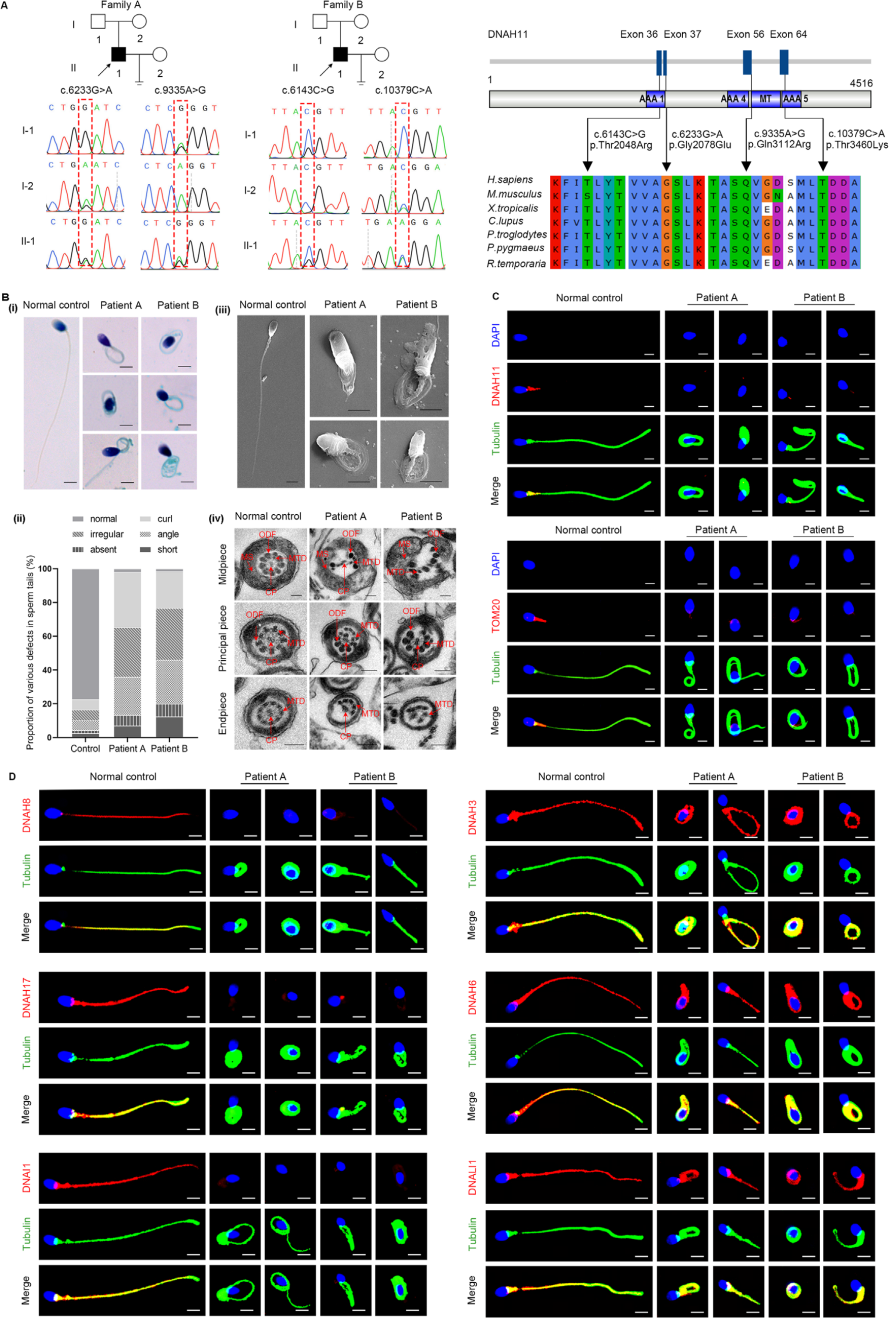
Identification of biallelic pathogenic *DNAH11* mutations in two infertile patients with MMAF phenotype. (A) Family pedigree and Sanger sequencing of the probands. The black square indicates the probands among these family members. The red dotted rectangle indicates the identified mutations. Multiple sequence comparison of DNAH11 protein for different species; the black arrows indicate the location of these variants. (B) A typical MMAF phenotype was observed in the patients by Papanicolaou staining (i) and scanning electron microscopy (SEM) (iii) compared with the normal control (scale bars, 5 µm). The patients exhibited a nearly 100% proportion of sperm tail defects (ii). The sperm ultrastructure was observed by transmission electron microscopy (TEM) in normal controls and infertile patients (iv) (scale bars, 100 nm). CP, central microtubule; MTD, peripheral microtubule doublet; ODF, outer dense fiber; MS, mitochondrial sheath. (C) Immunofluorescence staining of DNAH11 and TOM20 expression in human spermatozoa from the control and the patients (scale bars, 5 µm). Red, DNAH11 and TOM20; green, α‐tubulin; blue, DAPI. (D) Immunofluorescence staining of ODA‐related and IDA‐related proteins in spermatozoa obtained from the controls and infertile patients. The expression of DNAH8, DNAH17, and DNAI1 was significantly downregulated in spermatozoa from patients compared with those from the controls; There were no significant differences in the expression of DNAH3, DNAH6, or DNALI1 in the spermatozoa of patients compared with the controls (scale bars, 5 µm). Red, DNAH8, DNAH17, DNAI1, DNAH3, DNAH6, and DNALI1; green, α‐tubulin; blue, DAPI.

Papanicolaou staining and scanning electron microscopy (SEM) analysis were carried out to evaluate the aberrant sperm morphology of the patients. Notably, the two patients presented a typical MMAF phenotype characterized by a combination of short, bent, coiled, absent, and/or irregular sperm flagellum (Figure 1B, i–iii). Moreover, the spermatozoa ultrastructure was analyzed by transmission electron microscopy (TEM). Compared with a normal sperm flagellum composed of a “9 + 2” axonemal arrangement from the control, the sperm flagella from the patients frequently exhibited the absence of central pairs (CPs), as well as disordered or missing peripheral microtubule doublet (MTDs) and outer dense fibers (ODFs) (Figure 1B, iv). The defects in mitochondrial sheath were also obvious in the sperm of the patients (Figure 1B, iv).

Notably, DNAH11 was expressed in the midpiece of the sperm tail of the control, while no DNAH11 signal was detected in the flagella of the sperm of the patients by immunofluorescence staining (Figure 1C). Compared with the control, the infertile patients exhibited diminished TOM20 signals by immunofluorescence staining (Figure 1C), indicating the defects of mitochondrial sheath in the sperm flagellum of the patients. Considering that *DNAH11* encodes a motor protein of the ODAs, we performed immunofluorescence staining to assess whether the deficiency of DNAH11 affected the expression of other ODA‐associated proteins. The results showed that in the control, DNAH8/DNAH17 and DNAI1, which correspond to the heavy and light intermediate chains of the ODAs, respectively, were primarily localized at the sperm flagella (Figure 1D). However, these proteins were almost absent in the spermatozoa of the patients (Figure 1D). We further found that the expression of key IDA‐associated proteins of DNAH3/DNAH6 and DNALI1 in the sperm flagella of the patients was consistent with that of the control, indicating that the IDAs were not directly affected by DNAH11 deficiency (Figure 1D). Taken together, these data suggested that DNAH11 deficiency causing male infertility with MMAF may be related to a defect in ODA assembly resulting from the reduced expression of ODA‐associated proteins.

In addition, intracytoplasmic sperm injection (ICSI) treatment was attempted for the *DNAH11*‐mutated patients. The basal hormone data of the female partners were normal and ovulation induction was performed before oocyte retrieval. In the ICSI cycle of the patient A's wife, six Day 3 (D3) blastocyst embryos were obtained after standard embryo culture. The couple then underwent the transfer of two blastocysts and ultimately achieved clinical pregnancy. Unfortunately, during the ICSI cycle in the partner of the patient B, five available D3 embryos were obtained, but the implantation failed after two embryos transferred. Collectively, we suggested ICSI as an optional treatment for patients with *DNAH11* variants and the failure of ICSI in patient B might be associated with other unidentified female factors.

Lucas et al previously demonstrated that the *Dnah11^iv^* mice, carrying a missense (E2271K) mutation in the AAA2 domain of Dnahc11 (the mouse homolog of DNAH1), displayed reduced sperm motility [3]. For humans, only two studies have reported the single nucleotide polymorphisms of *DNAH11* in patients with asthenozoospermia, suggesting that *DNAH11* mutations may be a risk factor for male infertility [4, 5]. However, the exact connection and the underlying mechanism between *DNAH11* mutations and sperm flagellar defects remain largely unknown in humans. Intriguingly, our study first identified two biallelic mutations in *DNAH11* in two unrelated infertile patients with an MMAF phenotype, who denied suffering from any symptoms of PCD. Further investigation revealed the downregulated expression of ODA‐associated proteins in the spermatozoa flagella of the patients. Taken together, our findings suggested that mutations in *DNAH11* might affect ODAs assembly in human sperm tail and further result in an MMAF phenotype.

Notably, previous studies showed that ultrastructural defects of the ODA were not detected in cilia of most PCD patients with *DNAH11* variants by conventional TEM [2]. However, the subtle ODA defects with abnormal ciliary beat patterns, hyperkinetic ciliary beating and reduced beating amplitude were visualized by high‐speed video microscopy in these patients [2]. Specifically, high‐speed video microscopy revealed a deficiency of >25% in the proximal ODA volume within the proximal ciliary region in *DNAH11* mutation patients [2]. Reasonably, in our study, obvious ODA defects were not detected in sperm flagella of the patients by conventional TEM. However, this does not imply the absence of subtle defects, which might be visualized using high‐speed video microscopy.

The clinical ICSI outcomes of MMAF patients have varied greatly due to differences in the pathogenic genetic etiologies. For example, successful ICSI outcomes for MMAF patients caused by mutations of *DNAH1*, *DNAH2*, and *DNAH8* have been reported [1]. However, MMAF men harboring mutations in *DNAH17* exhibited poor embryo quality after ICSI treatment. In this study, one patient with *DNAH11* variants achieved successful outcomes with ICSI, while another patient experienced poor ICSI results. Certainly, additional female risk factors should not be overlooked. Collectively, further studies are required to explore the relationship between the ICSI outcomes and *DNAH11* variants.

In conclusion, our study first identified two biallelic *DNAH11* variations as a new genetic factor of MMAF and further provided adequate genetic and therapeutic counselling for male infertility. More cases and further studies using knock‐in mice that mimic the *DNAH11* mutations found in humans to demonstrate the exact pathogenic mechanism between *DNAH11* variants and MMAF phenotypes are urgently warranted.

## Author Contributions

X. Y. performed the most of experiments. DM. L. and YH. Y. collected samples from human and collected the clinical data. GC. Z. conducted the clinical evaluations and wrote the original draft. GC. Z. and Y. S. conceptualization and reviewed the manuscript. Y. S. supervised the study. All authors critically reviewed and approved the final version of the manuscript.

## Ethics Statement

This study was approved by the Ethics Committee of West China Second University Hospital (2020053). All subjects gave informed consent to participate in the study before taking part.

## Conflicts of Interest

The authors declare no conflict of interest.

## Supporting information




Supporting Information

## Data Availability

The published article includes all datasets generated or analyzed during this study. The WES data supporting the current study have not been deposited in a public repository because of privacy issues but are available from the corresponding author on request.
