# Supplementary material for: Biallelic DNAH11 Variations Cause Male Infertility with Multiple Morphological Abnormalities of the Sperm Flagellum in Humans
Source: MedComm (2020). 2025 May 10;6(5):e70210. doi: 10.1002/mco2.70210 (PMC12064945; doi:10.1002/mco2.70210)
Supplement: Supplementary file 1 — Supporting Information [file MCO2-6-e70210-s001.docx]

**Biallelic *DNAH11* variations cause male infertility with multiple morphological abnormalities of the sperm flagellum in humans**

Xue Yang ^1,#^, Dingming Li ^2,#^, Yihong Yang ^1,#^, Guicheng Zhao ^2, ##^, Ying Shen ^1, ##^

^1^ Department of Obstetrics/Gynecology, Key Laboratory of Obstetric, Gynecologic and Pediatric Diseases and Birth Defects of Ministry of Education, West China Second University Hospital, Sichuan University, Chengdu, China

^2^ Department of Andrology/Sichuan Human Sperm Bank, Key Laboratory of Obstetric, Gynecologic and Pediatric Diseases and Birth Defects of Ministry of Education, West China Second University Hospital, Sichuan University, Chengdu, China

^#^ X.Y., DM.L. and YH.Y. contributed equally to this work.

^##^ GC.Z. and Y.S. should be consider Co-correspondence Authors.

**Correspondence:** Guicheng Zhao, Department of Andrology/Sichuan Human Sperm Bank, Key Laboratory of Obstetric, Gynecologic and Pediatric Diseases and Birth Defects of Ministry of Education, West China Second University Hospital, Sichuan University, Chengdu, 610041, China. E-mail: [gczhao1989@126.com](mailto:gczhao1989@126.com)

Ying Shen, Department of Obstetrics/Gynecology, Key Laboratory of Obstetric, Gynecologic and Pediatric Diseases and Birth Defects of Ministry of Education, West China Second University Hospital, Sichuan University, Chengdu, 610041, China. E-mail: [yingcaishen01@163.com](mailto:yingcaishen01@163.com)

**Materials and methods**

**Clinical Subjects**

Two infertile patients diagnosed with asthenoteratozoospermia and their parents were recruited from nonconsanguineous families in Sichuan Province, China. These two patients exhibit normal testicular volume and sex hormone levels. A thorough clinical examination revealed no identifiable infertility-related factors, including androgen or endocrine abnormalities, hypogonadism, cryptorchidism, varicocele, abnormalities of the vas deferens, or testicular tumors. Chromosomal analysis confirmed a normal male karyotype (46, XY) with no deletions detected in the azoospermia factor (AZF) region of the Y chromosome in the patients. After all the subjects provided signed informed consent, clinical data and peripheral blood samples were collected. This study was approved by the Ethics Committee of West China Second University Hospital. All studies were conducted in accordance with the Declaration of Helsinki.

**WES and Sanger sequencing**

A blood DNA mini kit (Foregene, DE-05112) was used to isolate genomic DNA from the peripheral blood of infertile patients and the family members for whole-exome sequencing (WES). The concentrations of all the genomic DNA were >50 ng/µl, the total amount of DNA was >3 µg, and the absorbance values (A260/280 nm) were between 1.8 and 2.0. Then, 1 µg genomic DNA was enriched for the human exome using the Agilent SureSelect Human All Exon V6 Kit, followed by WES sequencing on an Illumina HiSeq X System (150-bp read length). Further analysis was performed by collecting variants that met the following criteria: (1) variation in minor allelic frequencies ≤ 0.01 in any public database, including the 1000 Genomes Project, gnomAD, and ExAC browsers; (2) any nonsynonymous, frameshift, splicing, start loss, or stop-gain variations; (3) the variants were not predicted to be benign or likely benign. Additionally, the OMIM database (https://www.omim.org/) and relevant literature were examined to explore potential associations with male infertility. Assuming a recessive inheritance pattern, monoallelic variants were excluded from the analysis. Sanger sequencing was applied to validate the mutations detected by WES. The PCR primers used for the subjects and their family members are as follows: F1: 5′-GGACGACACTACAGGGACATGAT-3′ and R1: 5′-GAACGCCAATCCTCAAATTTCCTGC-3′; F2: 5′-CAGCCAGTGCACAAAGACTCTAT-3′ and R2: 5′-CACTACCTTACACTGAGTCAGGCC-3′; F3: 5′- GGCCCCTGACATTGAGCTAA-3′ and R3: 5′- TTTTAGACCCTGTGCCATGG-3′; F4: 5′- attgcttatgtgggggtggg-3′ and R4: 5′- TCCCTGTTGCTGGGGATCTA-3′.

**Papanicolaou staining and electron microscopy**

The fixed sperm samples were coated evenly on slides and air-dried for Papanicolaou staining to observe sperm morphology. Then, the samples were processed according to the instructions of a Papanicolaou staining kit (Leagene, DA0191) and observed under an upright microscope (OLYMPUS, CX31).

For scanning electron microscopy (SEM), sperm samples were fixed with 2.5% glutaraldehyde and coated on slides. The slides were gradually dehydrated with an ethanol gradient (30%, 50%, 75%, 85%, 95%, and 100%), and the specimens were dried and coated with gold particles using an ion sputter coater (Q150RS, RotaryPumped, Quorum Technologies). The samples were finally observed by SEM (S-3400, Hitachi).

For transmission electron microscopy (TEM), sperm samples were routinely fixed with 3% glutaraldehyde and 1% O_S_O_4_. After being embedded in Epon 812, ultrathin sections were stained with uranyl acetate and lead citrate. Finally, images were acquired by TEM (TECNAI G2 F20, Philips).

**Immunofluorescence staining**

The sperm samples were fixed in 4% paraformaldehyde solution, permeabilized with 0.3% Triton X-100 and blocked with 5% BSA solution at room temperature. After that, the samples were incubated with primary antibodies at 4 °C overnight, which included antibodies against DNAH3 (Cusabio, CSB-PA823461LA01HU, 1:100), DNAH6 (Proteintech, 30073-1-AP, 1:50), DNAH8 (Atlas, HPA028447, 1:200), DNAH11 (Bioss, bs-14360R, 1:50), DNAH17 (Proteintech, 24488-1-AP, 1:50), DNALI1 (Proteintech, 17601-1-AP, 1:50), DNAI1 (Proteintech, 12756-1-AP, 1:50), TOM20 (Proteintech, 11802-1-AP, 1:50) and alpha tubulin (Abcam, ab7291, 1:200). The next day, the samples were washed and incubated with secondary antibodies conjugated to Alexa Fluor 488 (Invitrogen, A11008, 1:800), Alexa Fluor 594 (Invitrogen, A11005, 1:800). Afterwards, the nuclei were counterstained with 4′,6-diamidino-2-phenylindole (DAPI, Sigma-Aldrich, D9542). Observation and imaging were performed using a laser scanning confocal microscope (Olympus, FV3000).

**Statistical analysis and data visualization**

Descriptive statistics were used to calculate the proportion of different defect types in the sperm tails, expressed as a percentage of defective spermatozoa to the total number of spermatozoa. Data were visualized using GraphPad Prism 8.4.0 software to generate bar graphs to illustrate the distribution of different morphologies of sperm between normal controls and patients.
